# Friction-driven scission: How nonlocal mechanisms contribute to membrane fission across domains of life

**DOI:** 10.1126/sciadv.adz7607

**Published:** 2026-01-07

**Authors:** Ane Landajuela, Carolina Gomis Perez, Patricia Bassereau, Andrew Callan-Jones, Erdem Karatekin

**Affiliations:** ^1^ Cellular and Molecular Physiology, Yale University, New Haven, CT, USA.; ^2^ Nanobiology Institute, Yale University, West Haven, CT, USA.; ^3^ Institut Curie, Université PSL, Sorbonne Université, CNRS UMR168, Physique des Cellules et Cancer, 75005 Paris, France.; ^4^ Laboratoire Matière et Systèmes Complexes, Université Paris Cité/CNRS (UMR 7057), Paris, France.; ^5^ Molecular Biophysics and Biochemistry, Yale University, New Haven, CT, USA.; ^6^ Wu Tsai Institute, Yale University, New Haven, CT, USA.; ^7^ Université Paris Cité, SPPIN – Saints-Pères Paris Institute for the Neurosciences, Centre National de la Recherche Scientifique (CNRS), F-75006 Paris, France.

## Abstract

Membrane fission is an energy-consuming process, critical for all domains of life. Prototypical fission machineries use local energy input such as nucleoside triphosphate hydrolysis to constrict and cut membranes. However, some membrane fission reactions paradoxically rely on protein scaffolds that by themselves stabilize rather than cut membranes. It turns out these proteins do not work alone; they use nonlocal energy input that generates a membrane tension gradient. Such a gradient mobilizes membrane flow that in turn tends to relax the membrane tension gradient. By interfering with membrane flow, the protein scaffold causes the membrane tension to increase unchecked to the point of mechanical failure, membrane fission. This friction-driven scission (FDS) mechanism is generic, conserved from bacteria to humans, and only requires two ingredients: a membrane tension generating process and a protein scaffold that hinders the associated membrane flow. Because both are often present in cells, it is likely that FDS contributes to membrane fission more frequently than previously appreciated.

## Teaser

A membrane fission mechanism uses a membrane tension gradient and a protein scaffold that hinders the associated membrane flow.

## INTRODUCTION

Membrane fission is essential for life (*1*). It is required for cell division for both prokaryotes and eukaryotes, enveloped virus replication, intracellular membrane trafficking, and other fundamental biological processes. Several membrane fission machineries have been identified and characterized (*1*–*4*), but important questions regarding mechanisms remain.

**Fig. 1.**
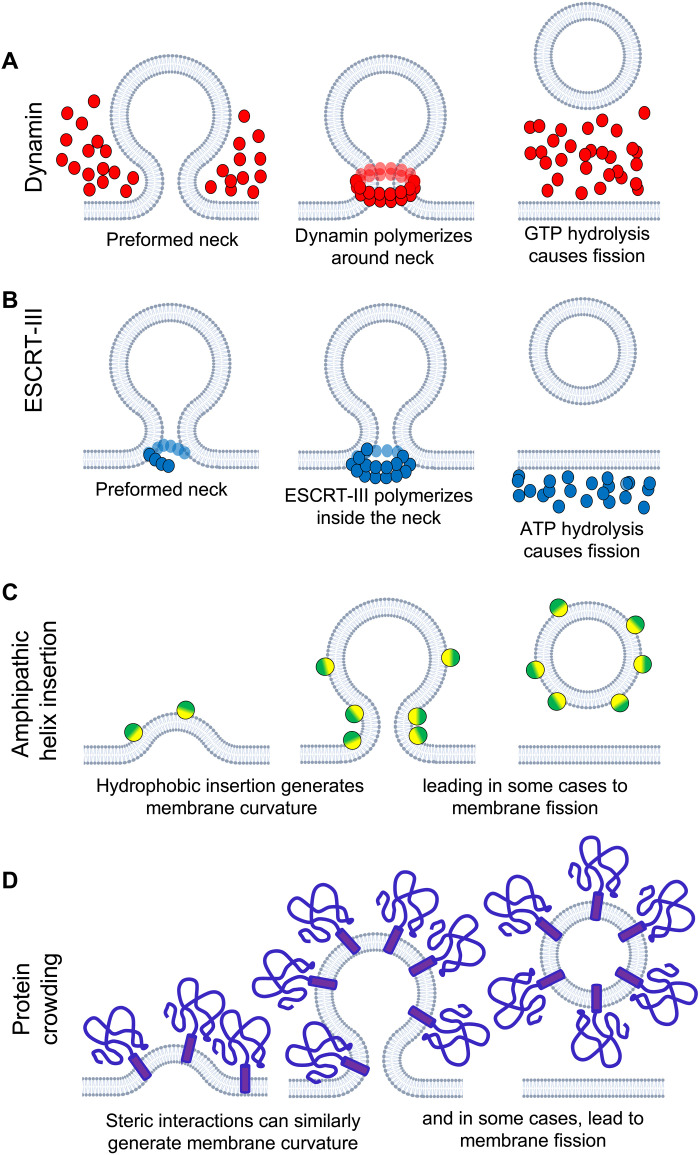
Membrane fission can occur through diverse mechanisms that appear unrelated. (**A**) Dynamin is recruited to membrane necks formed by other proteins during endocytosis in mammalian cells. It polymerizes into a helix around the neck and uses GTP hydrolysis to energize membrane fission. (**B**) The ESCRT-III machinery oligomerizes at the mouth of a bud facing away from the cytosol. ESCRT-III polymerization and/or ATP hydrolysis leads to membrane fission, but the mechanism is debated. (**C**) Interactions of proteins with amphipathic helices can lead to membrane budding and fission. Insertion of an amphipathic helix into one of the leaflets creates a mismatch between the surface areas of the two leaflets, which is relieved by curving the membrane. In some cases, the effect is strong enough to cause membrane fission. (**D**) Protein crowding (*9*) can lead to membrane budding and fission. If proteins are recruited at a sufficient density on one side of a membrane, curving the membrane provides more space to accommodate them.

Membrane fission involves changes in membrane shape and topology that can be described by membrane mechanics, but mechanics alone are insufficient to explain membrane fission in some cases. Proteins such as the ESCRT complex (*3*, *5*), FtsZ (*6*), or dynamin (*2*, *7*) form spiral-like filamentous complexes and use adenosine 5′-triphosphate (ATP) or guanosine 5′-triphosphate (GTP) hydrolysis to actively constrict membranes to promote their fission (Fig. 1, A and B). Other proteins use their free energy of binding and oligomerization to impose their spontaneous curvature on membranes. These include amphipathic helix-containing proteins such as epsin and endophilin (*8*) (Fig. 1C); proteins with disordered domains that deform and sever membranes by crowding (*9*) (Fig. 1D); and more generic spontaneous curvature-inducing proteins (*10*). However, in the cases discussed below, local membrane constriction alone by specialized proteins was found to be insufficient for inducing membrane fission, suggesting that additional processes must assist membrane fission in the cell.

One of these processes is the generation of a membrane tension difference between two membrane regions. The tension-generating system can be completely independent of the membrane fission machinery, and can even be located far away (~1 μm). Membrane tension gradients can be generated by molecular motors pulling on the membrane, DNA translocation, actin polymerization, or other active processes. A membrane tension gradient provides a driving force for membrane flow from low to high tension regions. Such flows, in turn, tend to relax the membrane tension gradient (*11*, *12*). By interfering with these flows, the membrane fission machinery causes the membrane tension to increase unchecked to the point of mechanical failure, i.e., membrane fission. We refer to this mechanism as friction-driven scission (FDS).

Before we delve into examples supporting FDS, we emphasize three important aspects of this principle. First, FDS can only be understood if membrane dynamics are considered. Previously, membrane mechanical energy has been considered extensively in membrane fission, but dynamics received little attention. Second, FDS accelerates membrane fission greatly, but fission can occur in its absence, albeit less efficiently (*13*–*15*), like the acceleration of a chemical reaction by a catalyst. Third, as pointed out above, the membrane tension generating system can be independent and located away from the membrane fission machinery, i.e., nonlocal energy input can serve as a critical facilitator for membrane fission.

Below, we will examine the evidence for FDS in three systems where it has been firmly established and consider additional situations where FDS is likely to play a role. We will also speculate about other cases where FDS may contribute to membrane fission in less obvious ways. Moreover, since well-studied membrane scission mechanisms involving constriction necessarily create regions of high resistance to membrane flow, it is possible FDS is a more widespread mechanism, even if only synergistically working with other pathways whenever membrane tension gradients and lipid-binding proteins are present.

## FDS ACROSS DOMAINS OF LIFE

### FDS during clathrin-independent endocytosis

FDS was originally discovered (*14*) in the context of scission of membranes decorated by BAR domain proteins (*16*). BARs have two well-known roles that long seemed incompatible. On the one hand, they play a role in membrane fission (*17*), suggesting that they somehow destabilize narrow membrane structures. On the other hand, they assemble into stable, highly curved scaffolds around membrane structures such as tubules (*18*, *19*). How can BARs stabilize membrane structures and cause their fission at the same time?

**Fig. 2.**
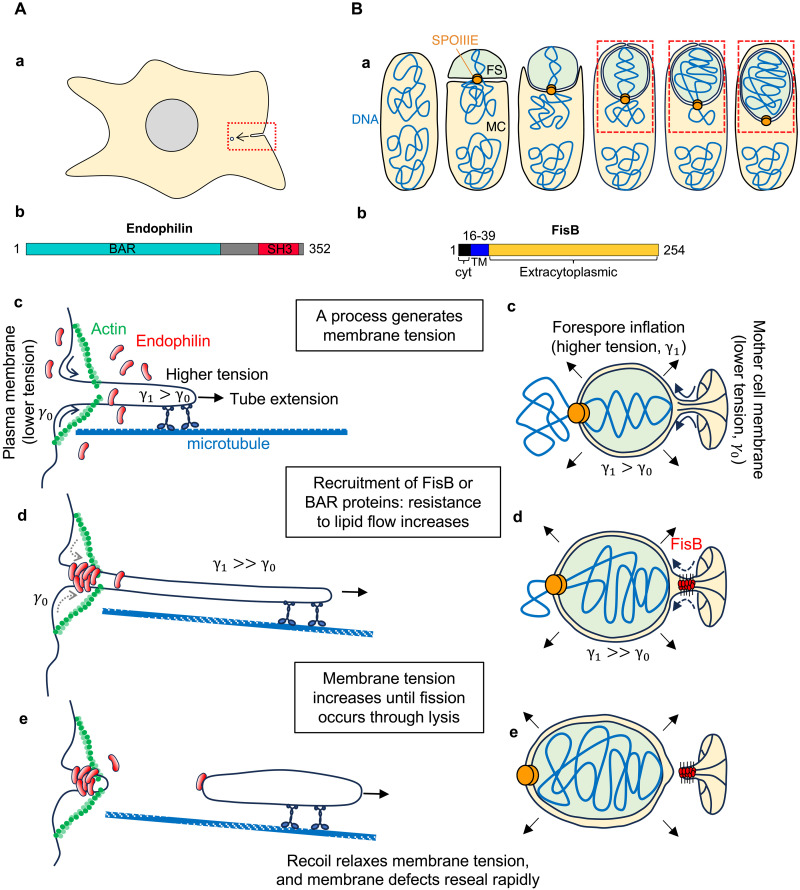
FDS during clathrin-independent endocytosis and in bacteria. (**A**) (a) Membrane tubes pulled from the cell membrane by molecular motors undergo BAR protein- and motor-dependent scission in mammalian cells (*15*). (b) Domain structure of Endophilin. [(c) to (e)] FDS during clathrin-independent endocytosis (*14*). Motors pull plasma membrane tubes toward the cytoplasm (c), generating a membrane tension gradient that drives membrane flow. Polymerization of endophilin around the tube impedes the flow (dashed arrows) (d), increasing tube tension further until fission occurs via lysis (e). To hinder lipid flow, the protein coat needs to be anchored to the plasma membrane or the cytoskeleton, or at least move slower than the growing tip of the tube, due to interactions with other components. (**B**) (a) In response to starvation, bacteria like *B. subtilis* form stress-resistant endospores. Following chromosome duplication, the bacterium divides asymmetrically, forming a small forespore (FS) and a larger mother cell (MC) compartment. The MC then engulfs the FS. Engulfment ends with a membrane fission event that releases the FS into the MC cytoplasm. After septation, most of the daughter chromosome is still in the MC and needs to be pumped into the small FS compartment using ATP hydrolysis, which inflates the FS compartment (*13*, *44*). Efficient membrane fission requires both FS inflation and a protein called FisB (*13*). (b) Domain structure of FisB. (c to e) Friction-driven membrane scission. FS inflation increases the tension of the surrounding membranes to near lysis values (*13*, *44*), driving lipid flow from the MC membrane through the membrane neck connecting the MC and engulfment membranes (c). By oligomerizing inside the neck, FisB impedes the membrane flow (d), causing the FS and engulfment membrane tensions to increase further, leading to membrane fission through lysis (e). Note that the topology is inverted compared to (A), as FisB acts from inside the membrane.

This paradox was initially explained by the proposal that BARs play only an intermediate role in membrane scission, such as in dynamin-induced membrane cutting, and were not themselves directly severing membranes (*20*, *21*). This view was challenged by the observation that endophilin, an N-BAR protein with amphipathic helices, could drive the vesiculation of large membrane vesicles (*8*). Yet, when this same protein was allowed to bind membrane nanotubes extracted from giant unilamellar vesicles (GUVs), it was found that it stabilized rather than cut them (*14*, *18*). Similarly, in cells undergoing clathrin-independent endocytosis, it was shown that, in the absence of dynamin, endophilin could cut membrane tubes, but this required action of dynein motors pulling and extending the tubes (*15*, *22*) (Fig. 2A). In vitro, endophilin-coated membrane tubes could be ruptured, but only by dynamic pulling of the tubes (*14*) that mimicked the extension of endocytic tubes by molecular motors. Before scission, increasing pulling speed increased the force to hold the tube (hence the membrane tension) as a result of protein-lipid friction; pulling a protein-free tube at the same speed resulted in no change in tube force and no membrane scission. Thus, increased membrane friction due to endophilin recruitment seemed to be the key to solving the paradox.

In the case of a protein-free tube, the tension perturbation generated by extending the tube is quickly relaxed by lipid movement from the GUV reservoir into the tube, sustaining its growth. The tube force does not exceed a few pN during pulling for rates up to ~10 μm/s, because the friction force remains small compared to the static force (measured at fixed tube length) that reflects a balance between competing tension and bending effects (*23*, *24*). Strictly speaking, this applies in cases where the GUV tension is controlled, e.g., by micropipette aspiration, or when a short tube is pulled. When a long tube is pulled from a GUV whose tension is free to adjust, the GUV tension and the tether force both increase substantially (*23*). Tube extension from tension-controlled, micropipette-aspirated GUVs showed that friction between the two sliding leaflets of the bilayer dominates at high pulling rates (*24*). Interleaflet sliding occurs because the extending tube removes more lipids from the outer leaflet of the GUV than the inner one. Nevertheless, even at pulling rates of ~200 μm/s the tether force remains small (<20 pN), and no membrane fission occurs (*24*). In experiments testing protein-lipid friction, extension rates are typically <10 μm/s and bare lipid bilayer tubes display little resistance to tube extension.

Simunovic *et al.* (*14*) found that, with endophilin-decorated membranes, the tube extension force was much larger than that observed with bare membranes, because protein-lipid friction impedes lipid flow. At the slowest extension rate of 20 nm/s, a very slow increase in the tether force was observed for 150 s, but no scission took place. At extension rates between 50 nm/s and 8 μm/s, 93% of trials resulted in scission. However, there seems to be no clear threshold for extension rates to cause scission, as tubes pulled by kinesin motors with speeds less than 50 nm/s also underwent scission. Note that, for friction to be effective, the membrane and the protein scaffold must slide against one another. This occurs, for example, if the protein scaffold is anchored to the cytoskeleton or the plasma membrane, while a tube is extended. Thus, the protein scaffold hinders membrane flows and isolates membrane tension changes at the two membrane compartments it separates. The tension increases on the side that is being pulled, until the tube undergoes scission (*14*). The friction force, and hence scission probability, could be modified through changes in interactions between proteins and lipids. Mutations that enhanced protein binding led to higher forces and facilitated scission; those that weakened binding led to lower forces and delayed scission (*14*).

How does increased membrane tension due to protein-lipid friction lead to membrane scission? A tube could be cut via an increase in membrane tension in two ways: The corresponding decrease in tube radius could facilitate scission by hemifission, as in the case of dynamin-mediated tube scission (*25*, *26*). The second way is through membrane lysis: appearance of a small pore that reseals rapidly with minimal leak as the membrane tension relaxes with the recoil of the scissioned membrane tube. For endophilin-mediated scission, it was concluded that the energy cost for hemifission was prohibitive, and that scission mostly occurred by pore formation in the tube (*14*).

The example of endophilin-scaffolded membrane tubes suggested a general principle whereby external pulling (i.e., by motors) of a tube scaffolded by any protein could result in scission because of friction. It was shown that centaurin, another BAR protein, could also coat tubes, despite having no amphipathic helices, and catalyzed scission upon dynamic extension (*14*). These observations first hinted at a principle in which a membrane tension increase, resulting from interference by membrane scaffolding proteins with tension-relieving lipid flows, could lead to scission.

### FDS by ESCRT-III proteins

Some protein complexes from the ESCRT-III family are implicated in the scission of membrane tubules in recycling endosomes, a crucial aspect of the endocytic pathway (*3*, *5*). Whereas these complexes are usually found in “reverse topology” (i.e., found inside the neck of membrane buds), the ESCRT-III subunits CHMP1B and IST1 can form scaffolds around the external side of membrane tubes (“normal topology”) (*27*–*29*). FDS can also occur here, but with the added twist of cross-talk between the scaffold proteins resisting lipid flows and the proteins extending the tubes. In addition to CHMP1 and IST1, a microtubule-severing ATPase called spastin is implicated in the cellular recycling of endosomal tubule-associated receptors (*30*). This led to the suggestion that spastin could directly cut endocytic tubules. However, a recent in vitro study showed that in the absence of spastin, CHMP1- and IST1-coated tubes were severed upon dynamic pulling at 3 μm/s, consistent with FDS, although the effect of pulling velocity was not systematically examined (*29*). Thus, spastin is not needed to sever tubes, but could contribute to scission in vivo through its interaction with microtubules, CHMP1B/IST1, or both. In vivo, scission by ESCRT-III complexes typically requires the VPS4 ATPase. In contrast, in this in vitro study, although Vps4 depolymerized ESCRT-III filaments, this did not lead to scission, which occurred only upon dynamic tether pulling. This suggests that scission of endosomal tubules with normal topology may be mediated by FDS independently of VPS4, although there is no evidence that it proceeds at slower pulling rates compatible with molecular motors. We also note that ESCRT-III and BAR domain proteins do not have any homology, supporting the idea that FDS might be a generic mechanism.

### FDS in bacteria

Bacteria remodel their membranes actively (*31*). In particular, they rely on membrane fission for every division cycle (*32*), for sporulation (*33*), shedding of minicells (*34*) or outer membrane vesicles (*35*), or the formation of internal vesicles (*36*, *37*). Yet, so far only one mechanism has come into focus for membrane fission in bacteria, during endospore formation (*13*, *38*, *39*). When starved, bacteria such as *Bacillus subtilis* produce dormant spores resistant to harsh environments. The first morphological event during endospore formation is an asymmetric division, producing a small daughter cell called the forespore (the precursor of the endospore) and a larger mother cell (*33*) (Fig. 2Ba). Thereafter, the mother cell engulfs the forespore, relying on peptidoglycan remodeling (*40*–*42*). At the end of engulfment, the mother cell’s membranes close and undergo fission, releasing the forespore, now surrounded by two membranes, into the mother cell’s cytoplasm. The mother cell then nurtures the development of the forespore into a mature spore and undergoes lysis to release it (*33*, *43*).

A protein named FisB was found to greatly increase the membrane fission efficiency at the end of engulfment (*13*, *38*, *39*). FisB has a short amino-terminal cytoplasmic tail, a single-pass transmembrane domain, and a ~200-residue extracytoplasmic domain (ECD) (Fig. 2Bb). It is conserved among all endospore forming bacteria, with no clear homology to other classes of proteins. Like eukaryotic BAR domain proteins or ESCRT-III components discussed below, FisB binds negatively charged lipids and oligomerizes at the site of membrane fission. Like for most ESCRTs, the topology is reversed: FisB ECD oligomerizes as trans-complexes inside the membrane neck that forms at the end of engulfment (*39*).

FisB ECD has no membrane curvature preference or ability to remodel membranes (*39*). How, then, does FisB oligomerization at the forespore neck promote membrane fission? A key observation was that in cells in which membrane fission was successful, the forespore volume was larger compared to cells in which fission did not occur, suggesting that forespore inflation was additionally required for efficient membrane fission (*13*). It is well-known that inflation occurs during engulfment (*44*), but it was not suspected that it could be linked to membrane fission. Forespore inflation is driven by DNA translocation into it by an ATPase, SpoIIIE, which increases turgor pressure inside the forespore compartment (*44*) and the tension of the membranes surrounding it to near lysis tensions (*13*). The tension perturbation drives membrane flow from the mother cell to the membranes surrounding the forespore, supporting the growth of the forespore surface area, until the recruitment of FisB to the membrane neck (*13*) (Fig. 2B, c and d). Continued DNA translocation into the forespore causes membrane tension to increase further as a result of FisB-generated friction, until membrane fission occurs (*13*) (Fig. 2Be). Like for BAR (*14*) and ESCRT (*29*) proteins, scission occurs at the boundary between the coated and uncoated membrane regions, because the entire FisB cluster is found either on the engulfment or the mother cell membrane after fission.

Consistent with this model, slowing DNA translocation (by using a slow-pumping SpoIIIE mutant) delayed forespore inflation and membrane fission, despite accumulation of larger copy numbers of FisB at the fission site. Conversely, in the absence of FisB, forespores inflated to large sizes but most failed to undergo membrane fission. Purified FisB ECD was found to impair lipid diffusion at high membrane coverage (*13*). When bound at high coverage, FisB ECD stabilized GUV membranes like BAR proteins (*13*).

A key property of membranes is that they are hardly stretchable: They lyse if stretched by more than a few percent area (*45*). Thus, membrane flow from low- to high-tension regions is essential to counter the membrane tension increase due to mechanical input and to prevent lysis. Impairing membrane flow by a protein scaffold leads to a membrane area deficit that can grow rapidly, even if the tension gradient driving lipid flux is small (*13*). For example, during FisB-catalyzed membrane fission, the lipid flux through the membrane neck connecting the mother cell and forespore membranes was estimated to be ~340 lipids/s (*13*). Assuming that the total area of the engulfment and forespore membranes is ~5 μm^2^, a deficit of ~300 lipids/s would cause a 1% deficit of the membrane area, i.e., 0.05 μm^2^, within ~4 min if the area expanded at fixed number of lipids, consistent with observed fission timescales. Thus, by interfering with lipid flux through the neck, FisB oligomerization leads to a further increase in the engulfment membrane tension and thus facilitates its scission.

A small fraction of *B. subtilis* cells successfully undergo membrane fission in the absence of FisB (*13*, *38*), but forespores are even larger in such cells, indicating their membranes are under higher tension (*13*). The presence of FisB makes membrane fission ~40 times more efficient (*13*). This suggests that even in cases when it is not absolutely required, protein-membrane friction could significantly increase the efficiency of fission.

## MEMBRANE FISSION AND PROTEIN SCAFFOLDS: IS IT FDS?

The above documented examples reveal that FDS can arise generically in distinct geometries and in different domains of life and does not necessarily require curvature-sensitive proteins. It occurs through the following sequence of events: (i) mechanical work generates a membrane tension perturbation, (ii) this, then, drives membrane flow, (iii) recruitment of a protein to the fission site acts as a frictional barrier, (iv) with reduced or no flow to relieve it, continued mechanical input increases membrane tension until fission occurs.

It is worth noting that the formation of a narrow membrane constriction such as a neck or tube is a prerequisite for membrane fission to occur, including via FDS. Less obvious, FDS occurs stochastically, and the loading rate (the rate at which membrane tension increases), friction, and timescales are interrelated. For example, at fixed membrane tube extension rate, fission is more likely to occur on a longer timescale and/or with higher friction. Conversely, with increasing extension (loading) rate the waiting time to observe membrane scission decreases (*14*, *46*). Similarly, with a short coat (low friction), a higher pulling speed or a longer waiting period increase the probability of observing fission. This is illustrated by FDS in *B. subtilis* during endospore formation where the coat size could be manipulated and quantified in vivo. Three hours after inducing sporulation, ~80% of cells expressing FisB at native levels undergo membrane fission, with ~40 FisB molecules accumulated at the fission site (*39*). When FisB expression is reduced, ~6 molecules at the membrane fission site, a shorter coat, cause fission in ~30% of the cells at the same time point (*39*). Even in the complete absence of a coat (Δ*fisb*), a few percent of the cells undergo membrane fission, but these are the ones whose forespores have grown fastest (higher loading rate). When forespore inflation is slowed (lower loading rate), only 40% of the cells undergo membrane fission, despite accumulation of ~80 FisB molecules at the membrane fission site (*13*). In addition to coat length, the total friction is determined by the intrinsic (per molecule) protein-membrane friction. This can be estimated by diffusion measurements of single proteins on lipid bilayers (*47*), but in practice may be difficult for some proteins. Computer simulations can be useful in this case (*48*). In summary, FDS has no strict requirement for a minimal coat size, loading rate, or timescale. In practice, the relevant timescales will likely be set by the rate at which FDS can cut a membrane, compared to other processes in cells.

Because of the variety of sources of mechanical work that can generate membrane tension gradients in the cell and the multiplicity of membrane-interacting proteins, it is natural to ask whether FDS is involved in other trafficking pathways. We summarize below instances where the necessary ingredients for FDS appear to be present, and suggest evidence that membrane scission is occurring through FDS.

### Scission of trans-Golgi network membrane tubes

In mammalian cells, specific cargoes are transported between the trans-Golgi network (TGN) and the endolysosomal system through clathrin-positive membrane carriers originating from TGN tubules. Dynamin isoforms, although detected on tubules, are not involved in this process (*49*, *50*). Instead, tubule cutting requires the following biochemical network. First, clathrin coat assembly initiates on flat membranes marked by phosphatidylinositol-4 phosphate [PI(4)P], sorting signals on cargoes, and the small GTPase adenosine 5′-diphosphate ribosylation factor 1 (ARF1) (*51*, *52*). Then, following conversion of PI(4)P to DAG, factors promoting actin polymerization are recruited, including the Rac1 GTPase, leading to tube extension. As this occurs, the BAR domain proteins arfaptin-1 and two form distinct patches near the tube base (*49*). Tubes are cut a few seconds after arfaptin recruitment; interestingly, the majority of scission events occur at the boundary between arfaptin-coated and arfaptin-uncoated regions. If arfaptin is deleted, the observed tubes are measurably longer and more abundant. This scenario may, however, be complicated by the fact that arfaptins interact with Rac1, and their deletion also affects actin polymerization (*53*).

Despite the complexity, the system contains the hallmarks of FDS: a mechanical input generating a membrane tension perturbation, this time via actin polymerization, and a protein scaffold that putatively resists lipid flow into the higher-tension tubule that would otherwise limit the increase in tension. There is in vitro evidence that these BAR domain proteins are recruited to tubules (*54*); however, dynamical information is missing, in particular, the effect of arfaptin on lipid diffusion remains to be quantified.

### Fragmentation of the endoplasmic reticulum

Up to here, we have described situations where FDS requires a protein scaffold. There is, however, at least one case where FDS can occur due to an accumulation of nonscaffolding, membrane-embedded proteins, and this occurs at interfaces between weakly and highly curved regions.

The endoplasmic reticulum (ER) forms a cell-wide membrane network that continuously undergoes tubulation, membrane fission, and fusion. It can change between a mostly tubular network to a sheet morphology in response to metabolic cues (*55*–*57*). The ER network is remodeled by proteins, such as reticulons (*58*) and atlastin (*59*), implicated in the fission and fusion of ER membranes. Like BAR domain proteins, reticulon was proposed to have seemingly contradicting roles: stabilization of high-curvature membranes (*58*, *60*) and helping their scission (*61*).

The *Drosophila* reticulon Rtnl1 was recently found to facilitate fission of extending ER tubules, both in *Drosophila* and when ectopically expressed in COS-7 cells (*46*). When reconstituted, reticulon coated artificial lipid tubes pulled from beads covered with proteo-lipid bilayers, but was unable to sever them unless the tubes were dynamically extended (no scission of lipid bilayers alone were observed under similar extensions) (*46*). When present, Rtnl1 caused high viscous resistance to tether formation and extension. Thus, friction caused by reticulon Rtnl1 was responsible for membrane scission (*46*).

The authors suggested increased friction caused membrane scission via hemifission based on the following observations. First, at high pulling speeds, the estimated tube radii were below 5 nm, close to the hemifission threshold (*46*). Second, increasing the fraction of negative curvature lipids that inhibit pore formation but promote hemifission, increased the fission probability. Modeling suggested that feedback between the curvature-driven Rtnl1 sorting toward the nanotube and tube constriction during elongation could amplify tube constriction, causing scission via hemifission (*46*). However, tube elongation rates and pulling force ranges used were very similar to those used by Simunovic *et al.* (*14*) in their study of endophilin-driven fission via lysis. Thus, it is possible that reticulon-mediated ER membrane fission involves contributions from both the lysis and hemifission pathways. In either case, membrane fission by Rtnl1 seems to rely on friction.

### Yeast endocytosis

In contrast with mammalian cells, where dynamin is known to be crucial for clathrin-mediated endocytosis (CME), in yeast the role of dynamin or dynamin-like proteins in CME is less central (*62*, *63*). Actin polymerization provides the force to deform and elongate clathrin-coated vesicular buds. Once the buds have reached roughly 50 nm in depth (~15 s after bud initiation), the N-BAR domain proteins Rvs161 and Rvs167, homologs of amphiphysin, are recruited near the bud base (*64*), and over the course of a few seconds, a scaffold forms along most of the bud length (*65*). These proteins form oligomers around the bud (*65*, *66*). Once Rvs161/167 have bound the membrane, the bud extends in a tubular manner up to about 150 nm in length before being cut into oblong vesicles (*64*). In cells in which these proteins are deleted, scission efficiency drops by about 30% (*67*); the invaginations that are cut are rounder and shallower (*64*). In vitro, Rvs161/167 and other yeast endocytic BAR domain proteins form stable scaffolds on PI(4,5)P_2_ containing membranes and strongly impair lipid mobility (*68*). These results suggest that endocytosis and scission can occur in the absence of any specific scaffolding by BAR domain proteins, and that the role of these proteins is more complicated than in the previously discussed examples. The late recruitment of Rvs161/167 and the size and shape of the scissioned vesicles suggest that they might play a role in the size regulation of endocytic vesicles as opposed to a strict requirement for scission. Nevertheless, the prerequisites for FDS are satisfied: (i) mechanical input creates a membrane bud; (ii) the resulting tension perturbation drives lipid flow into the growing invagination, supporting tube extension at ~20 to 30 nm/s (*65*, *69*); (iii) upon recruitment, the Rvs 161/167 forms a scaffold covering the entire tubular part of the invagination in about a second, resulting in a large frictional surface; (iv) actin polymerization causes the tension in the invagination to increase without the relaxing effect of facile lipid flows.

In addition to Rvs161/167, other membrane- and actin-bound proteins, such as myosin 3/5 and actin nucleators (*69*), could also impede lipid flow, giving rise to high tension along the bud, possibly explaining the modest loss of fission efficiency in the absence of Rvs161/167. It is therefore possible that FDS is occurring, but other mechanisms may predominate or act synergistically, such as neck constriction (*70*, *71*), as could be caused by the interplay of turgor pressure and actin-driven bud growth (*72*). It is currently difficult to disentangle the precise mechanism leading to scission; future measurements, including ones that probe lipid diffusion barriers in the bud and/or those that reconstitute the scission machinery in vitro (*73*), would help to clarify the role of FDS.

In mammalian cells, FDS may possibly explain puzzling observations by Xu *et al.* (*74*) on synaptic vesicle endocytosis at the calyx of Held synapse. GTP hydrolysis by dynamin is sufficient to mediate membrane fission in vitro, in the physiologically relevant case where dynamin oligomerizes just a few rungs on a membrane tube in the presence of GTP (*7*). Thus, while contribution of other factors has been suggested in cells (*7*), including clathrin polymerization (*75*), there is no compelling reason to suspect a role for FDS under normal dynamin function. Xu *et al.* (*74*) blocked endocytosis at the calyx of Held synapse by inhibiting dynamin using the nonhydrolyzable GTP analog GTPγS, or the guanosine 5′-diphosphate (GDP) analog GDPβS. Remarkably, continued stimulation and exocytosis accumulated vesicle membrane at the cell surface and restored endocytosis in the presence of the dynamin block. It is known that dynamin assembly in the absence of hydrolyzable GTP leads to long dynamin coats that stabilize membrane tubes rather than cutting them both in vitro (*76*–*79*) and in vivo (*80*). Similarly, a GTPase-deficient dynamin mutant results in long, dynamin-coated membrane tubes connected to the plasma membrane (*81*). Unexpectedly, GTP addition to preformed coats leads to membrane fission only in the presence of increased membrane tension (*76*). Thus, if the long membrane tubes coated with blocked dynamin were pulled, e.g., by actin polymerization, it is possible FDS would result.

### Mitochondria

In cells, mitochondria proliferate by division, as they cannot be generated de novo (*82*, *83*). While many of the molecular players in mitochondrial fission are established, the physical mechanism remains unclear. We briefly describe the sequence of events leading to fission. First, the fission site is established through a tight contact between the outer mitochondrial membrane and an ER tubule which wraps around the mitochondrion (*84*). In a second step, the ER tubule constricts the future mitochondrion fission site; this is thought to be powered by different mechanisms, perhaps working in parallel. Notably, formins (INF2) on the ER surface coordinate with the nucleator Spire1 on the mitochondrial surface to polymerize actin filaments causing a radial pressure-like force on the contact site (*85*). Furthermore, myosin 19 motors, recruited to the mitochondrial membrane, anchor ER-connected actin filaments, furthering constriction (*86*). Myosin II has also been implicated in actin-mediated constriction of the ER-mitochondrion contact site (*87*). These mechanisms reduce the mitochondrial diameter at the fission site from over 200 nm down to 100 nm (*85*). After this initial constriction, the dynamin-like protein Drp1 (Dnm in yeast), is recruited (*88*), and through GTPase activity causes a second constriction event, right up to fission (*89*, *90*). Drp1 recruitment and GTP hydrolysis to otherwise protein-free reconstituted membrane tubules is not sufficient to generate fission, either by constriction or by tubule extension (*91*).

Along these lines, Mahecic *et al.* (*92*) found that mitochondria constricted by the fission machinery do not always divide. The frequency of scission events correlated positively with mitochondrial membrane tension associated with cytoskeletal activity, although a membrane tension gradient could not be detected using a fluorescence lifetime probe (*92*). Knocking down actin and microtubules, and thus reducing the forces they apply directly or indirectly to the membrane, resulted in lower tension and lower rates of scission. It was proposed that increased membrane tension lowers the energy barrier for the constriction to shrink down and undergo scission. We suggest, however, that membrane dynamics might also be involved in the scission process. Namely, cytoskeletal forces compressing or pulling the mitochondria are expected to create membrane tension gradients. In addition, membrane tubes were observed to be pulled out of a mitochondrion asymmetrically (*92*) (on one side of the constriction). These would create membrane tension differences across the constriction site. With tightly apposed ER and accumulated proteins, including Drp1, the constriction site could act as a barrier to lipid flow and tension equilibration. Supporting this idea, the mitochondrion diameter is observed to be smaller on the side from which a tube is pulled. The asymmetric tension should also be manifested by an asymmetric recoil after fission: The higher tension part should recoil more strongly. To see if this were the case, we analyzed a subset of the time-lapse recordings Mahecic *et al.* (*92*). Of 15 cases picked at random, 6 had a difference between the two diameters of >100 nm. In all six cases, the thinner part recoiled strongly after fission, while the thicker part had limited recoil (data and details can be found at: https://data.mendeley.com/datasets/pzx6bbr257). Thus, cytoskeleton activity may lead to membrane tension gradients that cannot relax because of hindered lipid flow across the constriction site. As a result, it is plausible that FDS contributes to membrane fission.

## CONCLUSIONS AND OUTLOOK

**Fig. 3.**
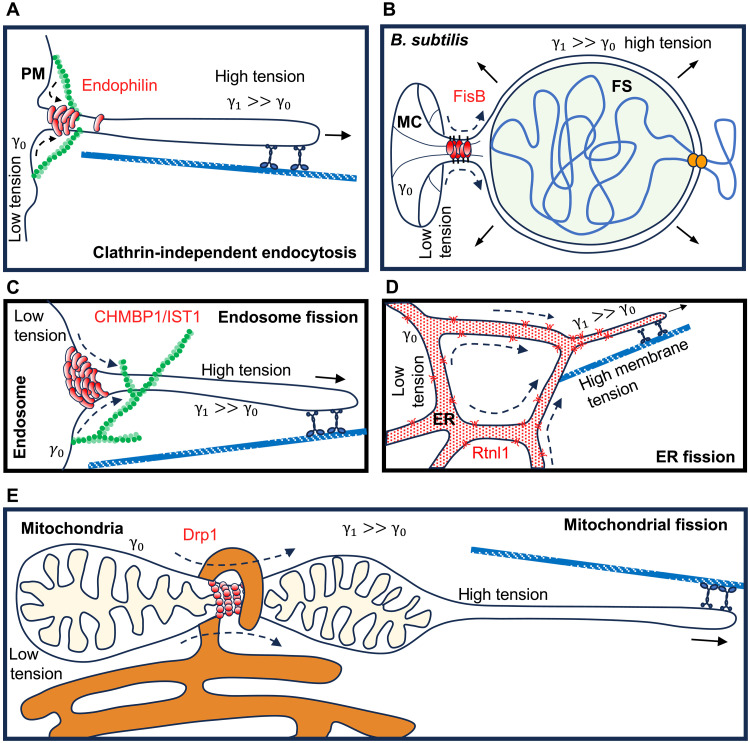
FDS may contribute to membrane fission widely. (**A**) FDS during clathrin-independent endocytosis. A membrane tension gradient is established by molecular motors pulling a membrane tube from the plasma membrane. Oligomerization of BAR domain proteins such as endophilin on the tube hinders flow of membrane into the tube (*14*) (see Fig. 2A). (**B**) FDS during endospore formation in *B. subtilis* (*13*). The membrane tension gradient is generated by an ATPase that pumps DNA into the small FS compartment, increasing turgor and inflating it (*44*). FisB forms a cluster inside the neck connecting the MC and the engulfment membranes, hindering membrane flow (*13*) (see Fig. 2A). (**C**) FDS by ESCRT-III components. Similar to endophilin (A), ESCRT-III components CHMBP1/IST1 oligomerize around membrane tubes hindering lipid diffusion, and can only drive membrane scission in the presence of additional forces pulling on the tubes (*29*). (**D**) Possible contribution of FDS to fission of ER tubes. This process requires reticulon Rtnl1 and external pulling forces on membrane tubes, neither of which is sufficient to cause fission alone (*46*). This suggests that hindering of membrane tension equilibration by Rtnl1 contributes to membrane scission (*46*). Reticulon does not seem to form dense aggregates on membranes, yet it increases viscous resistance to membrane tube extension (*46*). (**E**) Possible contribution of FDS to mitochondrial fission. This process requires Drp1, which by itself is able to oligomerize around membrane tubes and hinder lipid diffusion, but is unable to cut them. Additional forces are required for membrane fission, which are likely provided by motors pulling tubes from mitochondria (*92*). Dashed arrows indicate hindered membrane flow.

It is now established that FDS occurs in at least three distinct systems, using evolutionarily unrelated proteins and occurring in different domains of life: during clathrin-independent endocytosis using BAR domain proteins (*14*, *15*) (Fig. 3A), endosomal sorting using ESCRT-III components (*29*) (Fig. 3C), and endospore formation in *B. subtilis* using FisB (*13*) (Fig. 3B). We suggest that FDS may contribute to membrane fission more widely, because its ingredients are generic and present in many cellular contexts (Table 1 and Fig. 3, D and E).

**Table 1.** Membrane fission systems in which FDS has an established or plausible role. Bold row headers are cases in which FDS has been shown to be the mechanism underlying membrane fission. The rest are cases in which some key hallmarks of FDS are present, so FDS may contribute, but establishing this would require additional measurements, some of which are indicated in the comments column.

Membrane fission protein and reference	Process	Hallmarks of FDS			Comments
		Mechanical work leading to membrane tension gradient	Evidence for lipid flow	Evidence for increased resistance to membrane flow upon protein recruitment	
**Established role for FDS**					
**Endophilin A2 (EndoA2) (*14*) Fig. 2A**	Clathrin-independent endocytosis (CIE)	Microtubule motors (*22*).	In vivo, growth of membrane tubes from the plasma membrane (*15*).	In vitro, EndoA2-coated lipid tubes pulled from GUVs have decreased lipid mobility and increased resistance to extension (*14*).	First demonstration of FDS. EndoA2 forms a scaffold around membrane tubes pulled from GUVs that impedes lipid diffusion. EndoA2 oligomerization alone around a fixed-length membrane tube was insufficient for membrane fission. Only dynamically extended, EndoA2-coated tubes underwent membrane fission, for extension velocities 0.05–8 μm/s.
**CHMP1B-IST1 (*29*) Fig. 3C**	Tubular endosomal traffic from the ER	Microtubule motors (*122*).	In vivo, growth of membrane tubes from endosomes.	In vitro, CHMP1B-IST1 forms a scaffold around a tube pulled from a GUV that impedes lipid mobility and resists tube extension (*29*).	Similar observations and conclusions as for EndoA2-mediated membrane fission. EndoA2 and CHMP1B-IST1 are structurally unrelated, hinting that FDS may be a generic mechanism. Membrane fission was observed for tubes extended at 3 μm/s, but the effect of the pulling rate was not examined systematically.
**Fission protein in bacteria (FisB) (*13*) Fig. 2B**	Bacterial endospore development	Forespore (FS) compartment inflation due to DNA pumping by the ATPase SpoIIIE (*44*).	Growth of membranes surrounding the FS at the expense of mother cell membrane area.	In vitro, FisB forms a scaffold that acts as a lipid mobility barrier; in vivo, mother cell area decrease is reversed after membrane fission (*13*).	Showed FDS to be a generic mechanism across domains of life. FisB scaffolding and FS inflation were both required for efficient membrane fission. In the absence of either component, membrane fission still occurred, albeit much less efficiently. This suggests FDS can be an accelerator even in cases when it is not absolutely required.
**Likely role for FDS**					
Arfaptin (*49*)	Biogenesis of secretory granules at the TGN	Actomyosin network (*49*).	In cells, growth of membrane tubes from the TGN.	Not tested.	Clathrin-coated TGN tubules rely on actin and arfaptins, not dynamin, for scission. Although in vitro studies confirm arfaptin recruitment to tubules (*54*), dynamic data, specifically on its effect on lipid diffusion, is missing. In live cells, Golgi tubules are extended at ~0.6 μm/s (*123*). The system exhibits characteristics of FDS, but in vitro reconstitution is essential to clarify the roles of individual components.
Reticulon (Rtncl1) (*46*) Fig. 3D	Scission of ER membrane tubes	Microtubule motors (*124*).	In vivo, growth of membrane tubes from the ER.	In vitro, Rtncl1 increases resistance to tube extension (*46*). In vivo, Rtncl1 overexpression induces slower retraction of tubes (*46*).	Reticulon modifies the membrane rheology without forming a scaffold, increasing resistance to tube extension. Only dynamically extended membrane tubes containing reticulon underwent scission, consistent with FDS, for extension velocities 0.1–8 μm/s.
Rvs161/167 (*68*)	Yeast endocytosis	Actomyosin network	In vivo, growth of membrane tubes from the plasma membrane.	In vitro, Rvs161/167 forms a scaffold that slows the lateral diffusion of lipids (*68*).	Actin polymerization drives the elongation of clathrin-coated buds, at ~20–30 nm/s, with Rvs161/167 recruited later to scaffold the membrane. While scission can still occur in their absence, it is markedly less efficient. FDS likely contributes, but in vitro reconstitution of the fission process will be essential to clarify the specific roles of each component.
Dynamin-related protein 1 (*92*) (DRP1) Fig. 3E	Fission of mitochondria	Microtubule motors (*125*), actomyosin network (*126*).	In vivo, membrane tubes are pulled out of the mitochondrion asymmetrically (on one side of the constriction) (*92*).	The close apposition of the ER and the accumulation of protein complexes at the fission site, including Drp1, suggest frictional resistance to lipid flow, although this has not been tested directly.	Super-resolution imaging showed DRP1-positive constriction sites can revert and the fission machinery disassemble. Mitochondria that underwent successful fission had higher membrane tension (assessed by a fluorescent probe, MitoFlipTR) than those that reverted. Motors and cytoskeleton dynamics deform mitochondria, increasing membrane tension transiently. Mitochondria move at 0.02–0.6 μm/s (*127*, *128*). MitoFlipTR did not detect membrane tension gradients across the constriction, but frequent difference in diameter on two sides of the constriction hints to a membrane tension gradient (see text).

The first ingredient, the generation of a membrane tension gradient, can result from the activity of molecular motors (*15*, *93*), DNA pumps (*13*), solute transporters and osmotic forces (*94*, *95*), and membrane trafficking (*96*), among others. Once the first ingredient is in place, the second one follows from first principles: such membrane tension gradients are a driving force for membrane flows that support the growth of the membrane compartment that is under higher tension. Membrane flow counters the tension increase. The third ingredient is that proteins that increase membrane flow resistance are recruited to the site of fission. Membrane flow can be impaired by increasing friction via assembling a scaffold at the fission site (e.g., BAR proteins), or modifying membrane rheology otherwise (e.g., reticulons). While most proteins involved in membrane fission preferentially bind highly curved membranes, this is not a requirement for FDS, as illustrated by FisB, which has no membrane curvature preference, yet is a prototypical FDS protein (*39*). After the recruitment of proteins hindering membrane flow to the membrane fission site, continued energy input increases membrane tension further. With low extensibility, the membrane undergoes scission under increased tension.

How does membrane scission occur in FDS? Increased membrane tension can facilitate membrane fission in at least two ways, via hemifission or lysis. For a membrane tube pulled from a quasi-flat surface, as in the case of tubes pulled by molecular motors from the plasma membrane, increased membrane tension leads to a narrowing of the tube diameter (*97*). For a membrane neck connecting two flat surfaces, e.g., during endospore formation, increased tension also leads to neck thinning, but only if the neck is already narrower than a threshold (*39*). In either case, if the tube/neck diameter decreases below a critical value (*4*) (~5 nm), the inner leaflets merge (hemifission), followed by scission (*46*, *98*). Alternatively, scission can occur through lysis, without a hemifission intermediate (*14*). In this pathway, a small pore appears in the tube membrane and expands until the tube is cut. The tube ends reseal rapidly due to decreased tension while recoiling, with minimal loss of soluble contents (*14*).

When a protein scaffold is present on a membrane tube or neck, membrane scission almost always occurs at the scaffolded-bare membrane boundary. This is the case for membranes scaffolded by endophilin or centaurin (*14*), ESCRT-III components CHMP1B/IST1 (*29*), FisB (*13*), arfaptin (*49*), dynamin (*26*), and Rvs161/167 (*67*), consistent with scission through lysis. Why is this the case? The explanation may be rooted in the phenomenon of stress concentration near geometric or material discontinuities that is well-known in materials science (*99*, *100*). These discontinuities facilitate membrane rupture specifically at the scaffolded-bare membrane interface. Even in protein-free bilayers (*45*, *101*–*103*), tension-induced transient pores predominantly form through heterogeneous nucleation (e.g., due to impurities in the bilayer), as the energy barrier for homogenous nucleation is prohibitively high (*101*, *104*). In line with this, successive tension-induced pores in protein-free giant liposomes tend to reappear at the same site (*101*), and the presence of protein further facilitates pore nucleation (*14*). Thus, FDS elegantly repurposes the same proteins for multiple roles: recruitment to the membrane fission site, hindering of membrane flow, and nucleation of a membrane defect to initiate lysis.

Membrane flows occur commonly in cells, and often correlate with membrane fission activity. Why has not then FDS been implicated in more cases of membrane fission? The main reason may be that FDS couples long-range membrane dynamics with the local action of specific protein machineries. While the latter is a natural focus of mechanistic studies, the former is not, in part due to a lack of convenient tools and basic knowledge. Most past studies that considered the role of membrane dynamics in membrane fission focused on lipid and protein diffusion (*105*, *106*), local generation or consumption of lipids (*107*), membrane bending (*108*), or membrane flows (*109*–*111*) that occur on small length scales. Yet it is now emerging that many cell functions depend on coordinated, large scale flows in the cell membrane (*12*), including cell migration (*112*–*114*), exo-endocytosis coupling (*96*), axon growth (*115*) and branching (*116*), and patterning of protein distributions (*117*). Long-range propagation of membrane tension gradients on cell surfaces that drive such flows is an area of active research (*12*, *118*, *119*), with some cell types/regions displaying no measurable tension propagation (*120*), while others displaying rapid propagation (*96*, *116*). Membrane tension gradients are also present in endomembranes (*94*, *121*). While membrane tension gradient measurements in cells are challenging (*92*), changes of membrane compartment size, presumably driven by such gradients, can be more easily visualized and quantified (*13*, *15*, *64*). This type of analysis could potentially reveal if FDS contributes to membrane scission in cells.

Firmly establishing the contribution of FDS to membrane fission can be challenging, but its ingredients can be tested separately. How a protein scaffold affects lipid diffusion is informative about scaffold-lipid friction. Reconstitution of the scaffold on artificial membrane tubes and applying pulling forces would be ideal, but impractical in many cases due to technical challenges. One can also target the membrane tension generating mechanism, another generic feature of FDS. This was possible in the case membrane fission during endospore formation in bacteria, but could be more challenging in mammalian cells due to possible nonspecific effects. For example, interfering with kinesin activity to slow tube pulling may cause a wide range of effects and mask a specific role in FDS.

**Fig. 4.**
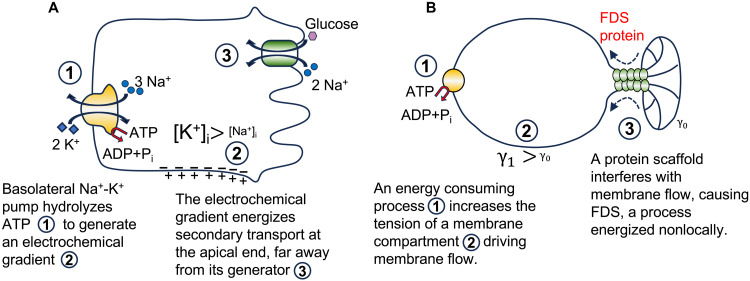
Analogy between secondary active transport and FDS, both of which rely on nonlocal energy input. (**A**) For every ATP molecule hydrolyzed, the Na-K pump extrudes 3 Na^+^ ions and imports 2 K^+^ ions (①). This generates K^+^ and Na^+^ concentration gradients across the cell membrane and contributes to the inside-negative membrane potential (②). The large electrochemical driving force for moving Na^+^ into the cell is used by secondary active transporters, such as the Na-glucose cotransporter (SGLT1), to move solutes up their electrochemical gradients (③). (**B**) An energy consuming process increases the membrane tension of a membrane compartment through osmotic or mechanical forces (①). This creates a membrane tension gradient (②) whose magnitude depends on how rapidly membrane can move from the low to high tension compartment. By interfering with such membrane movement, FDS proteins cause membrane tension to increase further, until membrane scission occurs. In this respect, they harness the nonlocal energy input that increased membrane tension to facilitate membrane fission.

FDS is reminiscent of secondary active transport processes, such as glucose uptake into a cell by the Na^+^/glucose cotransporter (SGLT), Fig. 4. SGLT uses the electrochemical gradient set by a primary active transporter at the plasma membrane, the Na-K ATPase. The Na-K ATPase is located basolaterally in most epithelial cells, where it hydrolyzes one ATP molecule to extrude three Na^+^ ions from the cytoplasm and to uptake two K^+^ ions. It is responsible for maintaining a low Na^+^ and a high K^+^ intracellular concentration. In addition, because a net positive charge is lost from the cell for each ATPase cycle, the Na-K ATPase is electrogenic, i.e., it contributes to the inside-negative membrane potential of the cell. The electrochemical gradient set by the Na-K ATPase is harnessed by SGLT, located at the other side of the cell, to move glucose into the cell against its electrochemical gradient. The energy penalty for glucose uptake is paid by the coupled transport of Na^+^ down its electrochemical gradient into the cell (Fig. 4A). In the case of FDS, a membrane tension gradient replaces the electrochemical gradient. SGLT cannot accumulate glucose inside the cell in the absence of a preexisting, favorable Na^+^ electrochemical gradient, set independently by an energy-consuming process (Na-K ATPase). Similarly, FDS proteins like FisB alone are inefficient to mediate membrane scission, unless an independent process creates a membrane tension gradient elsewhere, to pay at least partly for the energetic cost of membrane fission. Thus, like SGLT, FDS harnesses nonlocal energy input for its function (Fig. 4B).

FDS is based on a simple, energy-efficient principle, conserved across domains of life, suggesting that it may be an ancient solution for membrane fission that does not rely on highly specialized machinery. With the need for more specialized and more precisely timed fission events that arose during evolution, FDS may have become less critical for some membrane fission reactions. Still, because of its generic ingredients that are often present in cells, from bacteria to mammals, we suggest FDS may be more broadly contributing to membrane fission than appreciated.
